# Ultrafast Directional Janus Pt–Mesoporous Silica Nanomotors for Smart Drug Delivery

**DOI:** 10.1021/acsnano.0c08404

**Published:** 2021-03-06

**Authors:** Paula Díez, Elena Lucena-Sánchez, Andrea Escudero, Antoni Llopis-Lorente, Reynaldo Villalonga, Ramón Martínez-Máñez

**Affiliations:** † Instituto Interuniversitario de Investigacio′n de Reconocimiento Molecular y Desarrollo Tecnolo′gico (IDM), Universitat Politècnica de València, Universitat de València, Spain, Camino de Vera s/n, 46022 València, Spain; ‡ Unidad Mixta UPV-CIPF de Investigacio′n en Mecanismos de Enfermedades y Nanomedicina, Valencia, Universitat Politècnica de València, Centro de Investigacio′n Príncipe Felipe, 46012 València, Spain; § Unidad Mixta de Investigación en Nanomedicina y Sensores, Universitat Politècnica de València, Instituto de Investigación Sanitaria La Fe, 46026 València, Spain; ∥ CIBER de Bioingeniería, Biomateriales y Nanomedicina (CIBER-BBN), 28029 Madrid, Spain; ⊥ Nanosensors & Nanomachines Group, Department of Analytical Chemistry, Faculty of Chemistry, Complutense University of Madrid, 28040 Madrid, Spain

**Keywords:** Janus nanomotors, directional motion, ultrafast self-propulsion, drug delivery, on-command controlled release

## Abstract

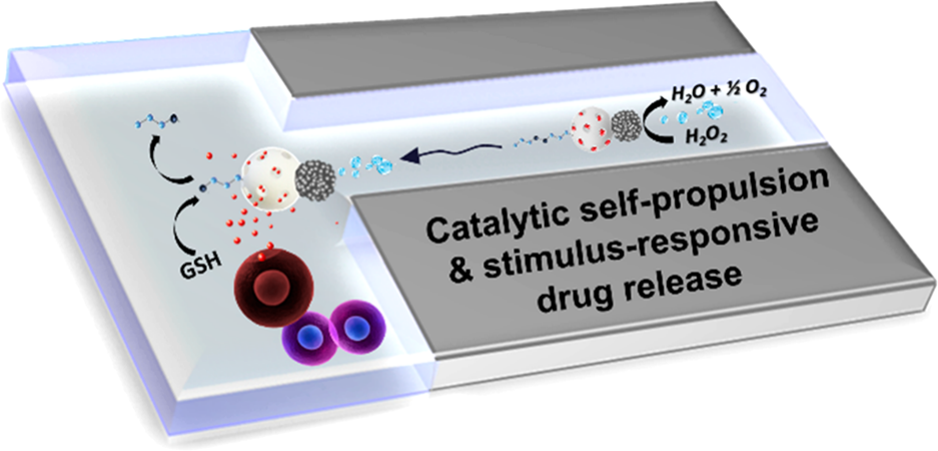

Development of bioinspired nanomachines with an efficient propulsion and cargo-towing has attracted much attention in the last years due to their potential biosensing, diagnostics, and therapeutics applications. In this context, self-propelled synthetic nanomotors are promising carriers for intelligent and controlled release of therapeutic payloads. However, the implementation of this technology in real biomedical applications is still facing several challenges. Herein, we report the design, synthesis, and characterization of innovative multifunctional gated platinum–mesoporous silica nanomotors constituted of a propelling element (platinum nanodendrite face), a drug-loaded nanocontainer (mesoporous silica nanoparticle face), and a disulfide-containing oligo(ethylene glycol) chain (S–S–PEG) as a gating system. These Janus-type nanomotors present an ultrafast self-propelled motion due to the catalytic decomposition of low concentrations of hydrogen peroxide. Likewise, nanomotors exhibit a directional movement, which drives the engines toward biological targets, THP-1 cancer cells, as demonstrated using a microchip device that mimics penetration from capillary to postcapillary vessels. This fast and directional displacement facilitates the rapid cellular internalization and the on-demand specific release of a cytotoxic drug into the cytosol, due to the reduction of the disulfide bonds of the capping ensemble by intracellular glutathione levels. In the microchip device and in the absence of fuel, nanomotors are neither able to move directionally nor reach cancer cells and deliver their cargo, revealing that the fuel is required to get into inaccessible areas and to enhance nanoparticle internalization and drug release. Our proposed nanosystem shows many of the suitable characteristics for ideal biomedical destined nanomotors, such as rapid autonomous motion, versatility, and stimuli-responsive controlled drug release.

Nowadays, one of the most ambitious objectives of nanotechnology is the development of advanced systems that mimic inherent functions of living entities, among which are autonomous movement,^1^ communication skills,^2^ and the ability to recognize and react to environmental signals.^3^ Regarding this topic, synthetic nano/micromotors have attracted much interest in recent decades. Research on these technology-based systems is focused on achieving efficient nanodevices capable of performing multiple “smart” tasks at the same time, such as responding to environmental stimuli and self-propelling.

Although there are numerous nano/microdevices powered by external forces, such as magnetic, acoustic, or electric fields, chemically powered prototypes are the most widely used. These motors’ autonomous movement is due to the conversion of chemical substances into mechanical energy by different transduction mechanisms, summarized in the generation of bubbles,^4−7^ auto-electrophoresis,^8,9^ or auto-diffusiophoresis.^10−12^ The main geometries that chemically self-propelled engines present are nanowires,^13^ microtubes,^14^ and Janus-type,^15^ whereas their composition is usually based on metals with intrinsic catalytic activity, among which platinum (Pt; that decomposes hydrogen peroxide in water and oxygen gas) and zinc (Zn; by Zn auto-oxidation to generate hydrogen bubbles) attract more attention. The propulsion mechanism followed by these active vehicles depends on their geometry but is more strongly influenced by their size, as at the nanometric scale the diffusion-type mechanisms govern the movement and any type of directionality is usually lost in favor of rotational diffusion.^16,17^ This is a drawback, as the implementation of this technology in real biotechnological applications, such as drug delivery, environmental bioremediation, or biosensing, usually requires controlling the artificial motors’ speed and direction toward a specific location. Furthermore, the most common and powerful man-made motors developed to date register high speed similar to biological motors, but they are driven by high concentrations of hydrogen peroxide (H_2_O_2_), being useful for nonbiomedical applications as sensor development, environmental bioremediation, and surface bacteria elimination.^18^ Nonetheless, lower levels of hydrogen peroxide can be found in human diseased regions, such as inflamed sites, infections, or cancer and cardiovascular diseases,^19^ which could be exploited by a nanomotor with low fuel requirements.^20^

In connection to biomedical applications for artificial devices, selective release of therapeutic payloads at their destination is highly desired. The advantage that nano/micromotors present compared to other passive release systems is their ability to self-propel and to display tissue penetration. This could be translated into an improvement in drug delivery efficacy to target tissues and cells, reducing side effects.^21−23^ A wide variety of nanomaterials have been developed for drug delivery applications, among which we highlight systems based on mesoporous silica nanoparticles (MSNs) due to their high load capacity, biocompatibility, and the large external surface that is easily functionalized with supramolecular assemblies to modulate the delivery of payloads.^24,25^ These capping systems provide devices with an intelligent response to specific environmental stimuli keeping a practically zero cargo delivery until the “opening signal” is recognized in the local environment. MSNs have been widely used as passive and “smart” delivery systems with excellent results, where the cargo is released from the nanopores through a simple diffusion process. However, these gated nanomaterials have practically not been tested for the development of chemical-powered nanomotors with controlled delivery capabilities.^26,27^ To achieve this goal, it is necessary to include an element that generates the driving power in the MSN. The few designs published to date generally integrate thin layers of catalytic metals by physical or chemical deposition on the silica support as a “motion system”, creating Janus-type anisotropic spheres, Table SI-1(i).^11,16,26−33^ This metallic layer considerably reduces the external surface of the silica, which is a great disadvantage to anchor the stimulus-responsive systems that provide the ability to respond toward the environment. Additionally, these designs require high concentrations of toxic fuel to record random Brownian motion through a self-electrophoresis or self-diffusiophoresis mechanism, making them usually incompatible for implementation on biological samples. More recent examples include highly active enzymes (such as urease and catalase) anchored to the external silica surface as a propellant element that generate the movement based on the enzymatic transformation of chemical substrates.^39−44^ Although, in these cases the fuel is not inconvenient, since they are self-propelled at low concentrations of biocompatible propellants, and the type of movement is still in some cases Brownian, making them difficult to control.

Based on the above, we report herein Janus-type Pt-MSN nanomotors as nanocarriers with multifunctional capabilities, such as catalytic self-propulsion and drug delivery in a specific and glutathione-mediated way for payload release in cells (Scheme 1). In order to provide Janus nanoparticles with improved autonomous motion, compared to typical Janus-type nanoparticles synthesized by electron beam evaporation or sputtering techniques, an innovative nanomaterial was designed based on a toposelective synthesis^45^ founded on a selective surface manipulation (at the Pt/ligand/MSN interface), using a Pickering emulsion where MSNs are partially exposed for the chemisorption of Pt nanodendrites (PtNds) by thiol bonds. In our proposed design, MSN nanoparticles are used as nanocontainers with a redox-sensitive gating mechanism, whereas the PtNds are employed as propelling elements to convert chemical energy into bubble propulsion by the catalytic reduction of H_2_O_2_.

**Scheme 1:**
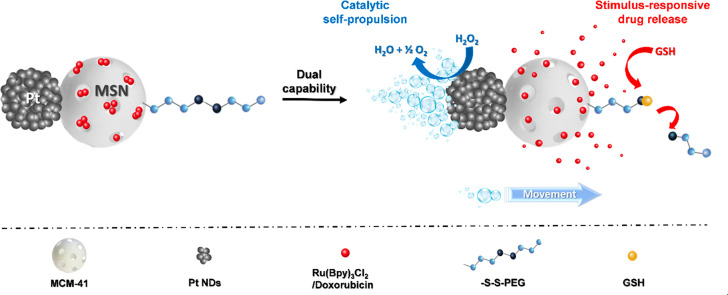
Schematic illustration of Janus Pt-MSNs nanomotors with a catalytic self-propulsion and a glutathione-mediated drug release.

PtNds were selected as motion systems, instead of using Pt layers, due to their major roughness and active catalytic area, which are expected to improve the propulsion capacity. In addition, this anisotropic nanomaterial has the advantage of maintaining most of the MSN face available for loading and effective external surface functionalization when compared to previous Pt-catalytic motors made by sputtering technology. Accordingly, we envisioned that nanomotors would be able to self-propel in the presence of low concentrations of hydrogen peroxide and would lead to an effective delivery of drugs once a reducing environment is reached in diseased areas. In fact, an ultrafast self-propelled motion and controlled drug delivery capabilities in THP-1 cancer cells, using a microchip device with physical limits, are demonstrated. The main features and innovation points of our system in comparison to previously reported Janus Pt-MSN nanomotors are summarized in Table SI-2.

## Results and Discussion

### Nanomotor Synthesis and Characterization

According to the synthetic procedure (SI-Scheme 1), Janus Pt-MSNs were prepared by the conjugation of two different nanoparticles in a single nanodevice.^45−47^ First and briefly, MSNs (calcined MCM-41-type, obtained by an alkaline hydrolysis reaction) were partially imbedded at the interface of the Pickering emulsion, formed by paraffin wax (oily phase) and water–ethanol (aqueous phase). The unmasked MSN surface was decorated with reactive thiol groups, by reaction with (3-mercaptopropyl)trimethoxysilane, on which PtNds were subsequently attached. After removing the paraffin with chloroform, Janus Pt-MSNs (**S**_**0**_) were obtained.

To synthesize the nanomotor, the PtNds in **S**_**0**_ was protected with 3-mercaptopropionic acid, and the mesoporous nanoparticles were loaded with the dye Ru(bpy)_3_Cl_2_ as a model cargo.^48^ The silica surface was further modified with (3-mercaptopropyl)trimethoxysilane, which was reacted with 2,2-dipyridyl disulfide^49^ and then with *O*-(2-mercaptoethyl)-*O*-methylhexa(ethylene glycol), allowing the capping of the mesopores with the redox-sensitive molecular gate (S–S–PEG) (**S**_**1**_). Another similar nanodevice was prepared using the cytotoxic drug doxorubicin as cargo (**S**_**2**_) for intracellular delivery studies.

**Figure 1:**
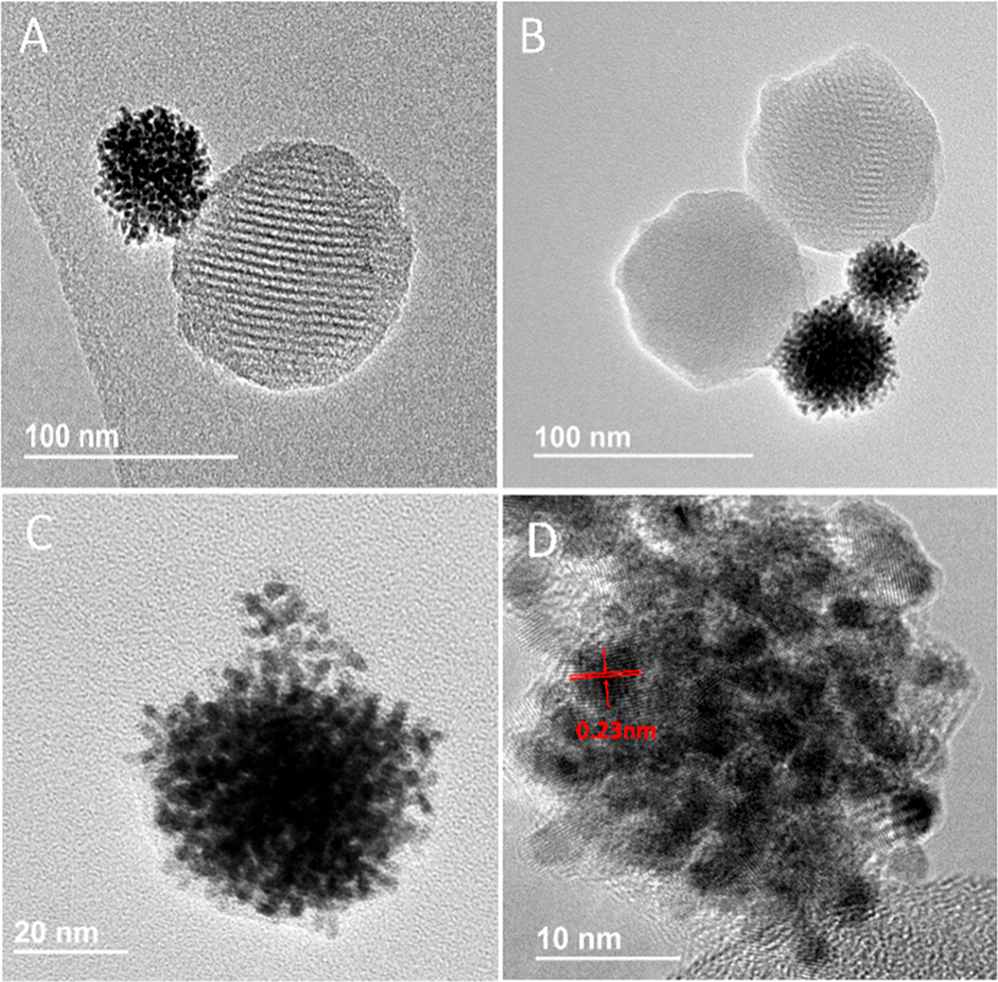
HR-TEM images of Janus Pt-MSNs (**S**_**0**_) (A, B) and PtNds (C, D), showing the interplanar spacing of 2.3 Å in the (111) plane of PtNd crystals.

To confirm the effective synthesis, nanomaterials were characterized using standard methods (see SI for details). The nanostructure morphology (snowman-like Janus) of the starting Pt-MSNs (**S**_**0**_) and PtNds was confirmed by high-resolution transmission electron microscopy (HR-TEM). Figure 1A,B (and SI-Scheme 1) shows the presence of PtNds attached to well-developed MSNs (100 ± 13 nm), confirming the successful synthesis of the anisotropic nanomaterial. PtNds (Figure 1C,D) are spherical in shape with an average diameter of 20 ± 9 nm. This spherical morphology is caused by the fusion of several Pt seeds of a size of 2.5 ± 0.3 nm. PtNds were synthesized by an autocatalytic surface reduction. In the synthesis of PtNds, the formed Pt^0^ sites by chemical reduction of H_2_PtCl_6_ (Pt^4+^) with l-ascorbic acid serve as catalytic sites for further reduction and atomic addition in the nanocrystal, resulting in a dendritic controlled growth controlled by absorption of a monolayer of polyvinylpyrrolidone (PVP) on the Pt surface. This mechanism produces a high increase in surface area and improves the PtNds’ catalytic capacity toward oxygen reduction.^50,51^ Furthermore, it is important to emphasize the high stability of PtNds that remained as nanodendrites even after PVP removal through heating and washing steps during the Pt-MSNs synthesis. Moreover, no apparent changes in PtNd size or morphology was observed after exposing the material to high concentrations of hydrogen peroxide for a long time (Figure SI-1).

HR-TEM analysis also revealed the presence of crystal faces in the synthesized PtNds with an average interplanar spacing of 2.3 Å (Figure 1D), which corresponds to the distance between the (111) faces in face-centered-cubic (fcc) platinum crystals.^52^ The crystalline structure was further demonstrated by the powder X-ray diffraction (PXRD) pattern showing four characteristic Pt diffraction peaks at high angles indexed as (111), (200), (220), and (311) planes (Figure SI-2). Moreover, the PXRD spectrum shows the characteristic Bragg peak of MCM-41-type structures at 2.63° (indexed as a (100) plane) in solids **S**_**0**_, **S**_**1**_, and **S**_**2**_, indicating that the mesoporous hexagonal network was not modified throughout the synthesis process, confirming the microstructure morphology observed by HR-TEM.

**Figure 2:**
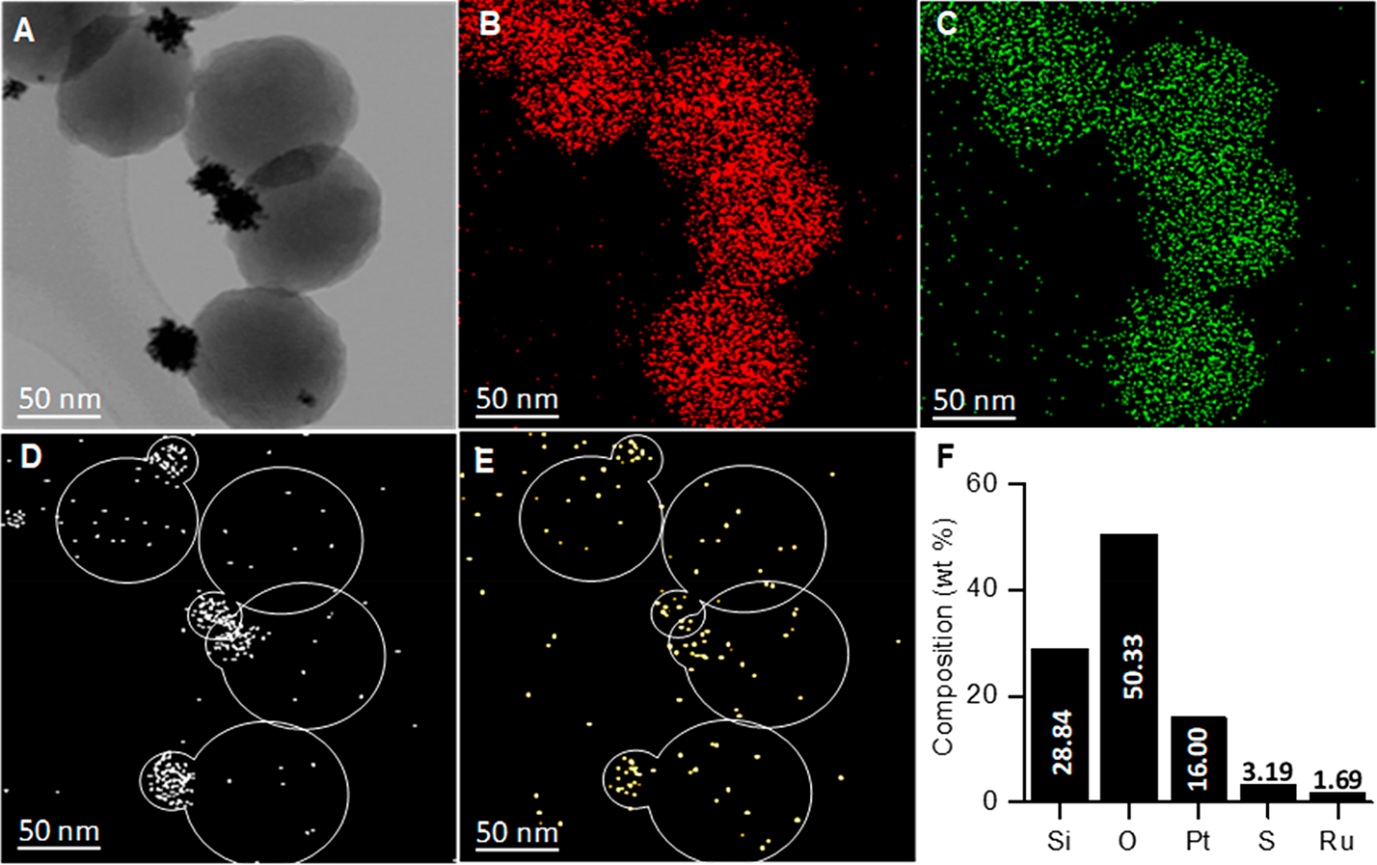
STEM image (A), STEM-EDX elemental mapping of Si atoms (B), O atoms (C), Pt atoms (D), and S atoms (E), and wt % composition detected (F) in **S**_**1**_ Janus Pt-MSNPs.

Additionally, the porous arrangement was corroborated by N_2_ adsorption–desorption isotherms of **S**_**0**_ and **S**_**1**_ (Figure SI-3). For **S**_**0**_, an acute adsorption process is shown at values of 0.1–0.3 *P*/*P*_0_ due to the condensation of nitrogen within the mesoporous network. However, this absorption step was not observed for the final nanodevice **S**_**1**_, which confirms the loading and capping of the pores. Pore size and specific volume and area for **S**_**0**_ and **S**_**1**_ were calculated (Table SI-3). The synthesized nanomaterial was also monitored by scanning transmission electron microscopy coupled with energy dispersive X-ray spectroscopy (STEM-EDX). The presence of Si, O, and Pt atoms of the Pt-MSNs scaffold is mapped in Figure 2B, C, and D images for **S**_**1**_, respectively. Moreover, Figure 2E and F demonstrated the presence of S atoms (assigned to disulfide-linked chains of the gatekeeper and the functionalization of the Pt surface) and Ru (attributed to the Ru(bpy)_3_Cl_2_ dye loaded in the nanochannels of the silica face).

Moreover, the content corresponding to the gatekeeper (S–S–PEG) and the loaded dye amounted 73.7 and 68.5 mg per gram of **S**_**1**_, respectively, which were obtained by thermogravimetric (Figure SI-4) and delivery studies. To complete the characterization, nanomotor assembly was evaluated by dynamic light scattering analysis (DLS) (Figure SI-5). The zeta potential showed a decrease in negative surface charge from −52.8 mV for **S**_**0**_ to −6.3 mV for **S**_**1**_, due to the immobilization of the redox-sensitive S–S–PEG gate and the loading with Ru(bpy)_3_Cl_2_ on the MSN face. On the other hand, the average hydrodynamic diameter increased from 145 nm to 335 nm for the initial and final nanodevices, respectively.

### Motion Analysis

Prior to characterizing the nanomotor motion, the catalytic activity of Pt in **S**_**1**_ was evaluated using a spectrophotometric assay based on the oxidation of 2,2′-azinobis(3-ethylbenzthiazoline-6-sulfonic acid (ABTS) in the presence of hydrogen peroxide in the 0–30 mM range. The reaction rate of ABTS oxidation (kinematically recorded by measuring absorbances at 415 nm) catalyzed by Pt in **S**_**1**_ at different concentrations of H_2_O_2_ showed a typical Michaelis–Menten behavior (Figure SI-6A). This demonstrated that the nanomotor exhibits a peroxidase-like activity, with an apparent affinity value (*K*_M_) for H_2_O_2_ that was calculated in 2.3 mM (from a Lineweaver–Burk plot according to equation-S1), a value that is comparable with native horseradish peroxidase (4.37 mM)^53^ (Figure SI-6B).

The self-propelled motion of the nanomotor **S**_**1**_ in PBS was characterized by nanoparticle tracking analysis (NTA) using a Nanosight NS300 instrument. In these experiments, the trajectories of nanomotors were recorded in real time in the absence and in the presence of different concentrations of the fuel H_2_O_2_, from 0% to 0.35% in buffer PBS solution. From each sample, five videos of 30 s (30 frames·s^–1^) length were registered and analyzed. The *x*–*y* coordinates of 20 nanoparticles (selected size between 100 and 200 nm) were extracted by the NTA 3.0 software, allowing the estimation of their mean square displacement (MSD) obtained with eq 1.

Motion studies were performed at short time intervals, Δ*t* < τ_r_ (rotational diffusion time or time required for the particles to move randomly, theoretically calculated in 0.8 s using eq 2), which means that the contribution of the ballistic displacement of fueled nanomotors is higher than the diffusive motion. At longer times, the ballistic trajectories of the nanomotors would be randomized again.^54^

**Figure 3:**
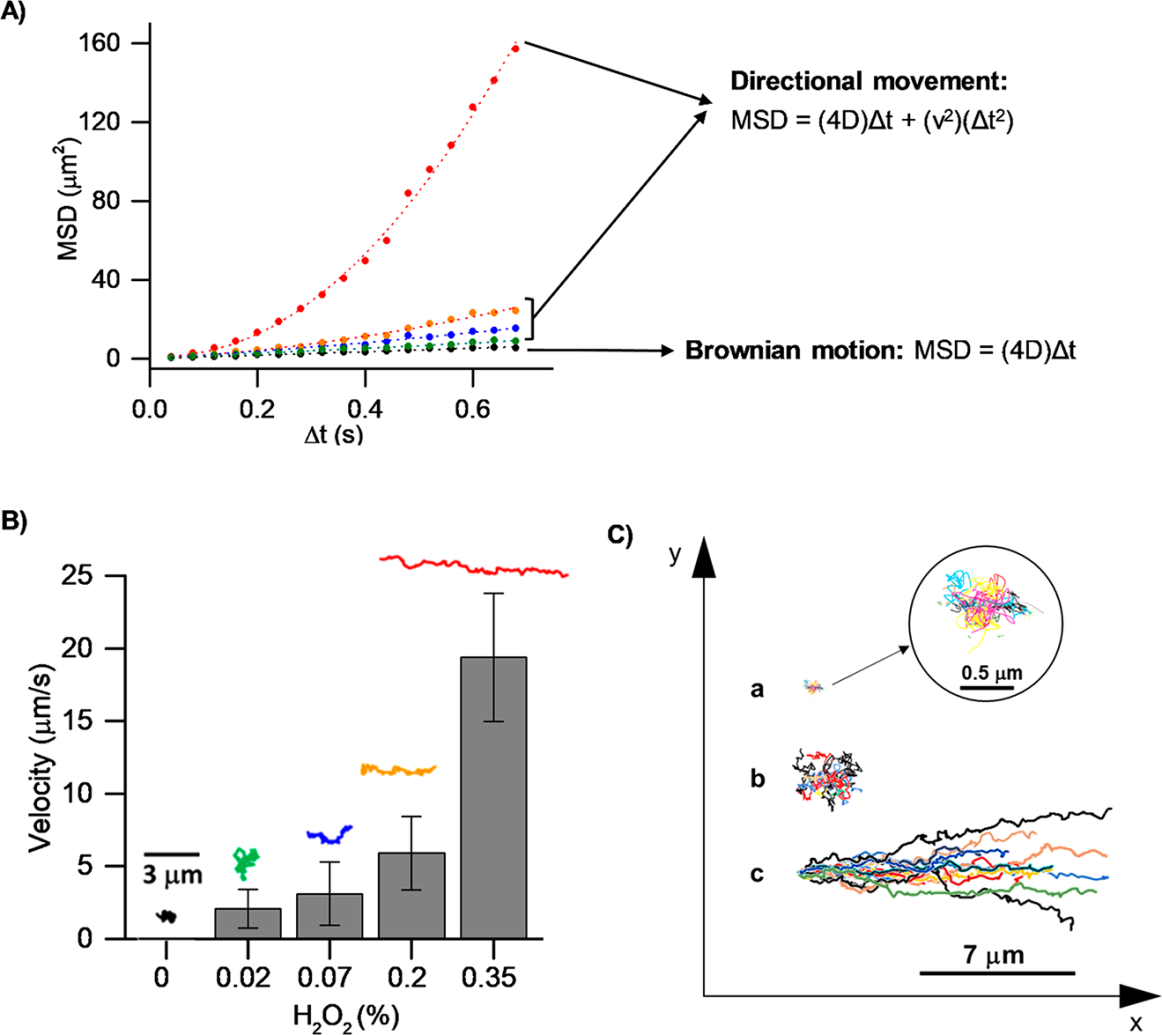
Motion characterization of Janus Pt-MSN **S**_**1**_ nanomotors. (A) Mean squared displacement as a function of time interval for the nanomotors at 0% (black), 0.017% (green), 0.07% (blue), 0.2% (orange), and 0.35% (red) fuel concentration. Curves were fitted using the equations shown in the figure. (B) Velocity of nanomotors at different fuel concentrations (*N* = 20; error bars represent the standard error of the mean velocities obtained). (C) Trajectories for 20 Janus Pt-MSN **S**_**1**_ nanomotors in PBS solution (a) and 0.07% (b) and 0.35% (c) hydrogen peroxide solutions.

After representing the calculated MSD values *versus* different time intervals (Δ*t*), the motion of nanomotors in each fuel concentration was evaluated (Figure 3A). When the movement is Brownian-type, MSD presents a linear correlation with Δ*t*. This diffusive behavior could be observed in the absence of fuel (0% H_2_O_2_). However, in the presence of H_2_O_2_ the MSD curves showed a parabolic shape, with an increase in the MSD values as a function of hydrogen peroxide concentrations, suggesting a directional movement due to a bubble-propulsion mechanism,^12,55^ which was corroborated by the appearance of visible bubbles at all fuel concentrations tested (Figure SI-7).

As observed in Figure 3B, the propulsion velocity, calculated with the parabolic MSD equation illustrated in Figure 3A, increased from zero to 19.4 μm·s^–1^ at 0 and 0.35% of fuel, respectively. This ultrahigh velocity supposes a relative speed of 149 body lengths (bl) per second, which, considering the concentration of fuel used, corresponds to a relative velocity of ∼426 bl·s^–1·^%^–1^. To our knowledge, this is the maximum speed reported for chemically powered Janus-type nano/micromotors and also for other nanodesigns, Table SI-1.^11,16,26−38^ Besides, that speed is even higher than that presented by natural mobile microorganisms such as *Escherichia coli* (8–15 bl·s^–1^).^56^ We attributed the ultrafast velocity shown to a catalytic enhancement due to the rough and large surface area that PtNds present, compared with conventional Janus micro/nanomotors made by chemical or physical vapor deposition of platinum.

Based on the velocities registered in Figure 3B, the propulsion force (*F*_prop_) applied on the nanomotors was calculated (by eq 3), which is equal to the repulsion or drag force (*F*_drag_) in our experimental conditions (at low Reynolds numbers).^57^*F*_prop_ values rose up to 53 fN with the increment of fuel concentration, supporting that the movement is governed by the ballistic force created in the decomposition of H_2_O_2_, leading to linear trajectories. Figure 3C shows the influence of fuel concentrations on nanomotor trajectories, monitoring a huge increase in the linearity of the nanomotor pathways at high fuel concentrations. The directional movement is promoted by the anisotropy of the nanomaterial,^12^ their high catalytic activity, and the location of the bubble growth. In our case it is expected that the center of the bubbles was always along the long axis of the dimer formed by the PtNDs and the MSN. Furthermore, the PtNDs’ rough surface participates in the stabilization of the bubbles once they nucleate,^55^ which can be observed with the naked eye after only 5 min of fuel addition (Figure SI-7).

Although activated nanomotors are propelled along a straight trajectory when the images were zoomed (in the *y*-positions), some Brownian contributions were clearly observed (Figure SI-8). Despite these reorientation movements, due to nanoparticle collisions with solvent molecules, the directional motion is retained.^59^ Lastly, the self-propelled motion was also evaluated by the translational diffusion coefficient (*D*_t_, calculated with the parabolic MSD equation recently mentioned), increasing its value up to 5.4 μm^2^·s^–1^ with the increment of fuel concentration (Figure SI-9). For nonactivated nanomotors, where the motion is controlled by Brownian diffusion, the diffusion value (*D*, extracted from a linear MSD equation) was 2.06 μm^2^·s^–1^. These results showed weak dependence of diffusion coefficients on the concentration of H_2_O_2_, in contrast with the velocity behavior.

It would have been interesting to record the positions of the nanomotors at higher time intervals to further characterize their motion, as recently published by Archer *etal*.,^58,59^ but it was impossible due to technical limitations (NTA provides trajectories of a maximum of 100 frames per particle) as well as the high speed exhibited by our nanosystems, quickly leaving the field of view. Although tracking of motors for longer times has been reported in previous studies using optical microscopes, it should be noted that nanoscale (<200 nm) objects are below the limit of resolution of optical microscopy.

### Controlled Release Studies

**Figure 4:**
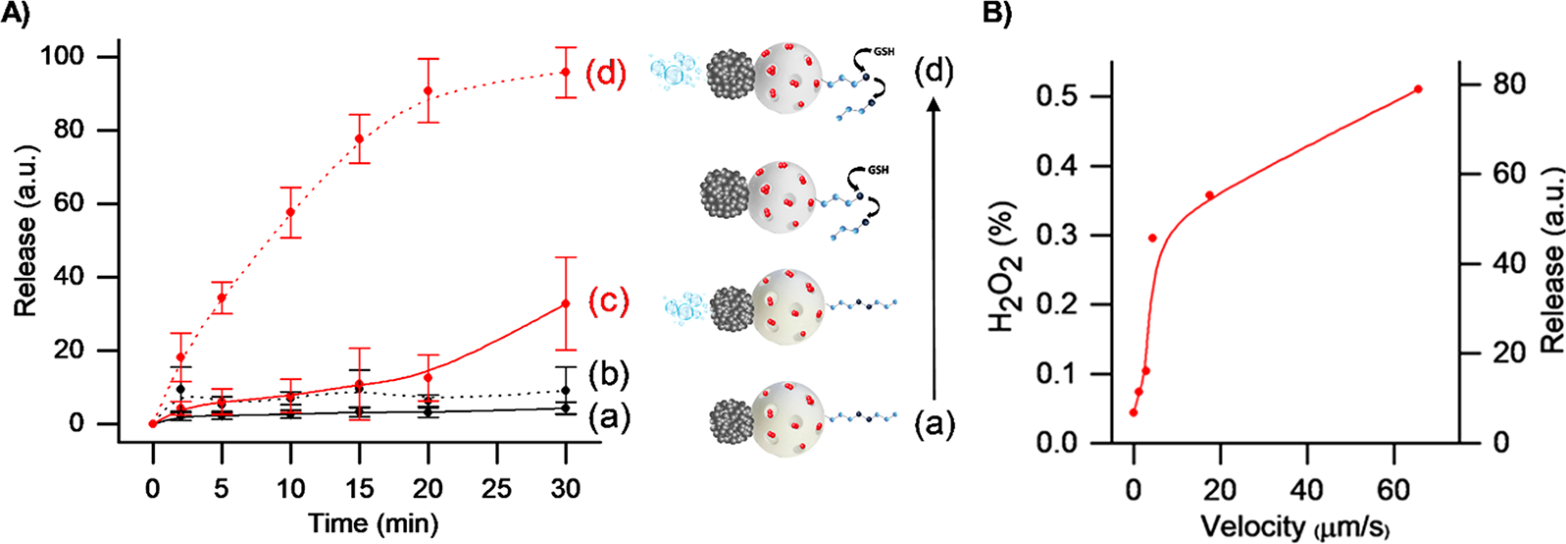
Normalized cargo release from **S**_**1**_ nanomotors in static conditions determined by measuring Ru(bpy)_3_Cl_2_ fluorescence (at 544 nm) *vs* time in aqueous solution (50 mM PBS pH 7.5), using (a) nanomotors without GSH and without H_2_O_2_ addition, (b) 0.1% H_2_O_2_-propelled nanomotors without GSH addition, (c) nanomotors with 10 mM GSH addition and without H_2_O_2_, and (d) 0.1% H_2_O_2_-propelled nanomotors with 10 mM GSH addition (A). Relation between H_2_O_2_ (%), velocities, and normalized cargo release (%) from **S**_**1**_ nanomotors (B).

Once directional motion of the nanomotor was confirmed, the ability of **S**_**1**_ to deliver the cargo under different conditions was evaluated. Release assays were carried out by suspending 0.5 mg of nanomotors in 1 mL of sodium phosphate buffer (PBS) in the presence and absence of reduced l-glutathione (GSH) at 10 mM (a typical reported intracellular concentration).^60^ GSH is the “key” molecule that triggers the opening of the capping system, releasing the cargo loaded inside the pores of the mesoporous face. Delivery studies were further conducted under static and stirring conditions and in the presence and absence of the fuel (0.1% H_2_O_2_) to evaluate the effect of the self-propulsion on the release of the Ru(bpy)_3_Cl_2_ cargo. Dye release kinetic profiles from **S**_**1**_ under static conditions are illustrated in Figure 4A. In the absence of GSH and H_2_O_2_, **S**_**1**_ remained capped and the delivery of Ru(bpy)_3_Cl_2_ was practically negligible (curve a). The same behavior was observed in the presence of H_2_O_2_, confirming that the gating system is not affected by the fuel (curve b). Moreover, a slow delivery kinetics from **S**_**1**_ under static conditions was triggered by the GSH *via* the rupture of disulfide bonds in the S–S–PEG gate (curve c). In contrast, in the presence of both inputs, H_2_O_2_ and glutathione, a faster release was observed due to the synergic effect of the self-propulsion and the uncapping of the nanoparticles by GSH (curve d).

This is supported by additional experiments that demonstrated that under stirring conditions cargo delivery from **S**_**1**_ in the presence of GSH and GSH + H_2_O_2_ was almost the same (Figure SI-10A). Besides, the influence of nanomotor velocity on cargo release was studied. For that, a dye delivery assay using **S**_**1**_ at a fixed time of 30 min, static conditions, and variable concentrations of fuel was performed. As observed in Figure 4B, an increase in nanomotor movement, as a consequence of higher fuel concentrations, results in an increment of cargo release to the medium. Finally, cargo release from nanomotor **S**_**2**_ (loaded with the cytotoxic drug doxorubicin) was also evaluated. A similar delivery behavior under static and stirring conditions in the presence of GSH and H_2_O_2_ to that found for **S**_**1**_ was observed (Figure SI-10B). The total amount of doxorubicin released was calculated spectroscopically and amounted to 0.7 μmol per gram of solid **S**_**2**_.

In summary, these experiments demonstrated that our Janus Pt-MSNs nanomotors display high self-propulsion capabilities at relatively low concentrations of H_2_O_2_*via* catalytic fuel decomposition. Moreover, the nanobots remain capped and only deliver their payload upon recognition of the reducing agent GSH, due to the cleavage of the gatekeepers. Thus, the nanodevice displays desired features for nanomotors in terms of biomedical applications: (i) autonomous motion and (ii) the ability to sense the environment and deliver the drug only in the presence of a stimulus (*i.e*., GSH).

### Self-Propulsion and Drug Release Capabilities in Cells

Nanomotors for biomedical applications should be propelled in the presence of low fuel concentration with no premature cargo leakage and deliver the payload once target cells or tissues are reached. Taking into account these concepts and encouraged by the above results (*i.e*., self-propulsion and controlled release capabilities), we studied doxorubicin delivery in THP-1 cancer cells by confocal fluorescence microscopy. In this experiment, THP-1 cells were incubated with a suspension of 50 μg mL^–1^ of **S**_**2**_ in the presence and absence of H_2_O_2_ (0.02%) at 37 °C for 30 min. Subsequently, uninternalized nanoparticles were removed by washing, and cells were further incubated for 1, 4, and 6 h in fresh medium. Before acquiring the confocal fluorescence image, the THP-1 cell nucleus and membrane were stained with Hoechst 3342 and WGA, respectively.

**Figure 5:**
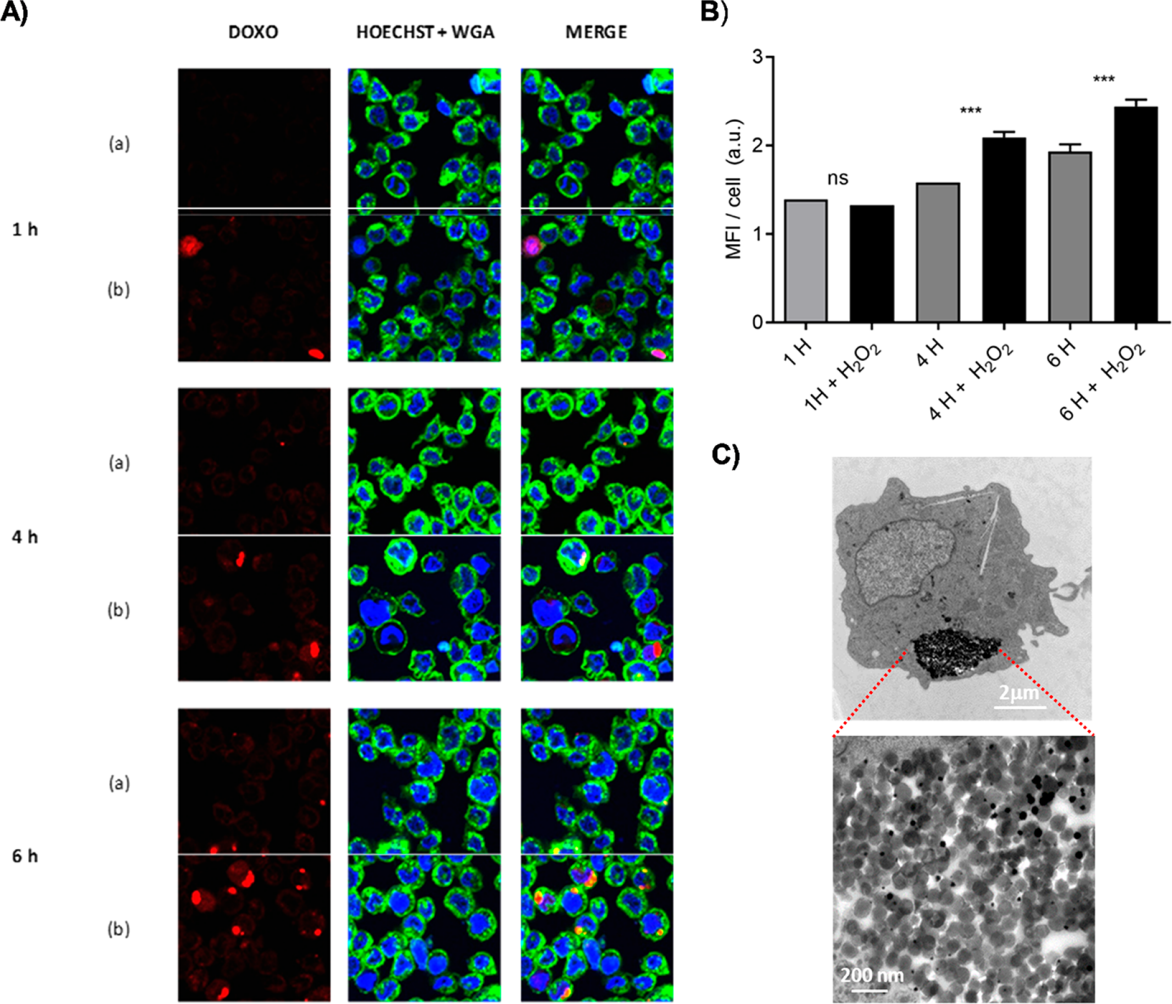
(A) Confocal microscopy images of internalization and controlled doxorubicin release of the **S**_**2**_ nanodevice in THP-1 cells at different times. THP-1 cells treated with 50 μg mL^–1^ of **S**_**2**_ nanomotors in the absence of fuel (a); THP-1 cells treated with 50 μg mL^–1^ of **S**_**2**_ in a medium containing 0.02% H_2_O_2_ (b). From left to right: doxorubicin fluorescence, DNA, and membrane fluorescence marker (Hoechst 3342 and WGA, respectively) and combined (merge). (B) Mean fluorescence intensity quantification in cells from confocal images at different times and with or without the addition of fuel. (C) TEM image of **S**_**2**_ nanomotor (100 μg mL^–1^) internalization in THP-1 cells after 6 h.

As shown in Figure 5A, an intracellular doxorubicin-associated fluorescence was observed indicating **S**_**2**_ internalization and the cleavage of the gatekeeper by intracellular GSH in both cases (absence or presence of fuel). Moreover, a larger intracellular doxorubicin release was observed for nanobots incubated with H_2_O_2_ after 4 and 6 h. This suggests that the enhanced diffusion of the nanobots in the presence of fuel results in an enhanced cell internalization and a larger intracellular cargo release. These results demonstrate the ability of the nanomotor to sense the redox environment in the cell and to specifically trigger the release of the cytotoxic drug. In addition, an improvement in cell uptake attributed to the autonomous movement of nanomotors was confirmed at very low fuel concentrations. Larger amounts of fuel are toxic for THP-1 and therefore were not studied.

Nanomotor internalization in cancer cells was also confirmed by TEM. Figure 5C shows the uptake of nanoparticles, and, as the image is zoomed, Janus-like nanomotor structures are clearly perceived. The results were completed with the WST-1 cell viability tests after 24 h, confirming that the concentration of **S**_**2**_ and fuel selected for the treatment carried out did not significantly affect THP-1 cells (Figure SI-11). Furthermore, a reduction in the amount of drug necessary to induce cell death by apoptosis was observed, noting that 200 μg mL^–1^ of **S**_**2**_ (corresponding to 0.14 μM of drug released) displayed the same cellular toxicity as 1.5 μM of free doxorubicin, which demonstrates the effective strategy of using fueled nanomotors to enhance drug delivery in cells for therapeutic applications.

Finally, as a proof of concept, the capacities of nanomotors mentioned above to perform autonomous movement and release therapeutic payloads in THP-1 cells were examined using a microchip device aiming to reproduce channels and fluids of living systems, such as blood vessels. Nanomotor testing on microfluidic platforms are the preapplication stage in more complex organ-on-a-chip devices to achieve future implementation in real therapies.^61^ Previous studies demonstrated how the use of this type of device with physical limits confines the space where nanomotors “swim”, controlling their speed and directionality without using external sources.^62,63^

**Figure 6:**
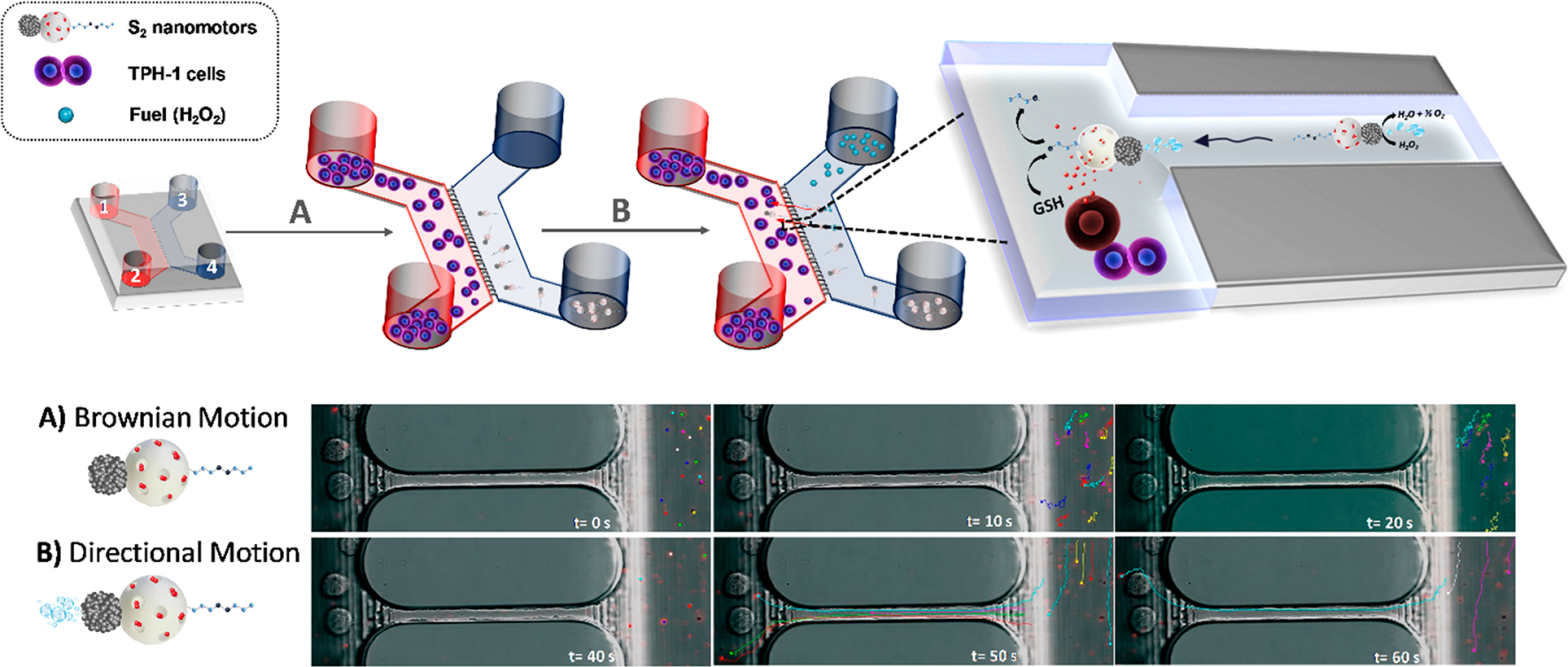
Three-dimensional scheme depicting the assay followed to study self-propulsion and drug release capabilities of **S**_**2**_ nanomotors in THP-1 cells using a microchip device and time-lapse optical images taken from Videos SI-1 and SI-2 of confocal microscopy showing the nanomotors’ motion behavior, which includes the trajectories analyzed by the Manual Tracking plug-in of the FIJI program, before (A) and after the addition of 0.02% H_2_O_2_ at 40 s of the experiment (B). (Experimental conditions: 10^8^ THP-1 cells mL^–1^ in wells 1 and 2, 0.02% H_2_O_2_ in well 3, and 100 μg mL^–1^ of **S**_**2**_ nanomotors in well 4).

For the assay, a four-compartment microchip (from XonaChips) was used, which allowed the compartmentalization of THP-1 cells, nanomotors, and fuel. The four wells are connected as 1–2 (red part) and 3–4 (blue part), and these were additionally connected to each other by micrometric channels (Figure 6). The experiment was performed in a cell culture media (RPMI-1640 buffer) depositing 108 THP-1 cells in wells 1 and 2, while 100 μg mL^–1^ of **S**_**2**_ nanomotors was added to position 3 (see Methods section for more details). Then 2 min videos were recorded by confocal microscopy (Video SI-1). Without fuel and after the addition of **S**_**2**_, the nanomotors diffused through the cuvette (blue part), and once stabilized, a Brownian motion behavior was observed, as shown in the trajectories recorded in time-lapse images taken from Video SI-2 (A).

At 40 s of assay time, the fuel (0.02% H_2_O_2_) was added to well 3, and then a clear directional movement was observed. Interestingly, this propelled some nanomotors through the microchannels toward the cells area (red part), as can be seen in the trajectories recorded from the Video SI-3 at different times (B). Activated nanomotors swam to the opposite side of fuel addition in a confined flow; the movement is denominated as negative rheotaxis. Velocities of **S**_**2**_ observed in that experiment seemed higher than the speed recorded by the NTA, when the cross section of the channel decreased from 100 μm to 4 μm (microchannels). This effect could be due to the bubble creation in a confined area and also to the enhanced flow velocity by a Venturi effect.^64^ The movement of the nanomotors through the microchannels to a wider area would mimic the effect suffered by circulating cells when moving from capillary to postcapillary vessels, with larger diameters, reducing their speed and moving toward the vessel walls. This process facilitates blood cells’ interaction with the endothelium and extravasation to tissues.^65^

**Figure 7:**
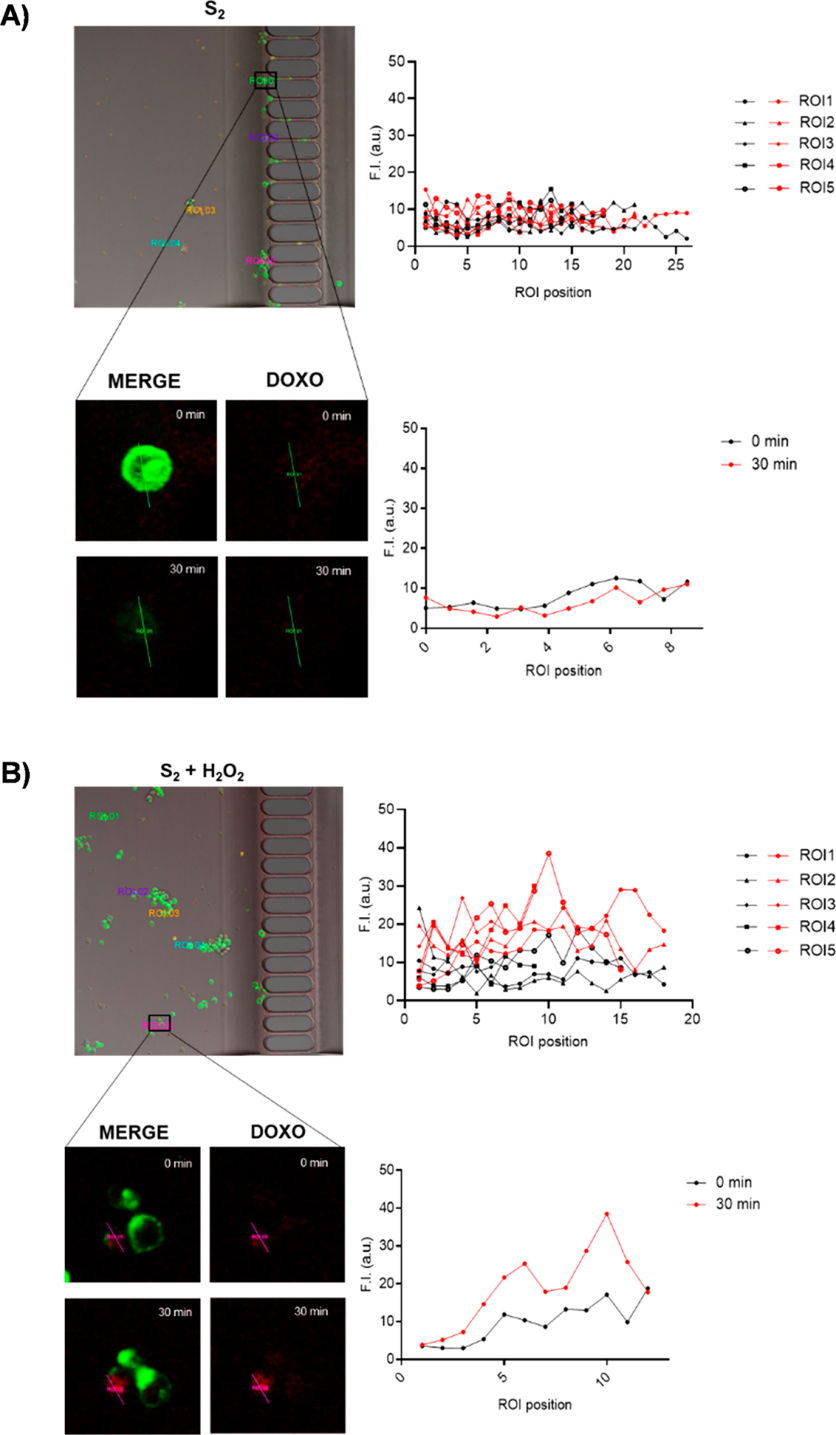
Image of THP-1 cells and **S**_**2**_ nanomotors (100 μg mL^–1^) in the microchip device in the absence (A) and the presence (B) of H_2_O_2_ solution (0.02%). Analysis of doxorubicin fluorescence intensity in five defined linear regions of interest (ROI 1–5), corresponding to different THP-1 cells at 0 (black) and 30 (red) minutes of test time (ROI 1 from **S**_**2**_ and ROI 5 from **S**_**2**_ + H_2_O_2_ zoomed).

Regarding controlled release of the drug, a similar experiment by confocal microscopy but extending the assay time to 30 min was carried out. Figure 7 shows the fluorescence intensities along the profile of several regions of interest analyzed, corresponding with different experiments (with and without the fuel). When H_2_O_2_-powered nanomotors reached cells through the microchannels, there was an increase in cell fluorescence over time, demonstrating the doxorubicin delivery was mediated by intracellular GSH. In the absence of H_2_O_2_, the fluorescence intensity was low and similar in both times analyzed, suggesting the inability of nonpowered nanomotors to cross microchannels and reach target cells, remaining in the channel between wells 3 and 4 (as observed in the Video SI-1). Note that nanomotors **S**_**2**_ remained capped and only delivered the cargo when internalized in cells.

## Conclusions

In summary, we report here the design, synthesis, and characterization of ultrafast and directional Janus Pt-MSNs nanomotors with stimuli-responsive drug release capabilities. They are based on the anisotropic conjunction of two individual nanoparticles with different and complementary functions: (i) a PtNds face, responsible for the autonomous self-propulsion *via* catalytic decomposition of H_2_O_2_ into water and oxygen bubbles and (ii) a redox-sensitive (S–S–PEG) gated-MSNs face, as a nanocontainer for the loading of the cytotoxic drug doxorubicin. The main advantage of PtNd nanoparticles used as a propelling element lies in their major roughness and high active surface catalytic area. PtNds endow an improved propulsion capacity to the nanomotors at very low concentrations of fuel, which was demonstrated by the elevated relative velocities registered (426 bl·s^–1^·%^–1^). These values are far superior to the speeds reported by previous chemically powered nano/micromotors reported, including Pt-catalytic motors made using sputtering technology, as well as by some mobile microorganisms. Moreover, the nanobots remain capped and only deliver their payload upon recognition of the reducing agent GSH, due to the cleavage of the gatekeepers. We also observed that an increase in nanomotor movement, as a consequence of higher fuel concentrations, results in an increment of cargo release to the medium. We also found that the nanorobot **S**_**2**_ is able to internalize in THP-1 cancer cells. THP-1 were selected as model cells to evaluate the selective drug release capacity of our nanomotors mediated by the reducing environment present in them. Moreover, a larger intracellular doxorubicin release was observed for nanobots incubated with H_2_O_2_ after 4 and 6 h. Finally, the use of nanorobots to target THP-1 cells was also studied using a microchip device with physical boundaries (microchannels), mimicking the transport along capillary vessels. The fast displacement of the propelled nanodevices facilitated nanomotors to pass through the microchannels toward the cells area, where they are internalized in THP-1 cells. However, nonfueled nanomotors were not able to reach target cells and deliver their cargo, revealing the advantage of using self-propelled nanoparticles.

Overall, our nanomotors possess highly desired properties for nanobots for biomedical applications such as autonomous motion, biocompatibility, efficient cargo transport, and stimulus-responsive controlled drug release in cancer cells. Future work is expected to delve into a more selective delivery of therapeutic payloads to target destinations, including specific cellular markers in the nanomotor design, as well as in other therapeutic strategies based on the elimination of hydrogen peroxide by their catalytic decomposition. It is widely reported that H_2_O_2_ produced by both cancer cells and associated fibroblasts causes DNA damage and inflammation, resulting in cell proliferation and metastasis. Besides, some resistance to tumor drugs is also associated with the presence of hydrogen peroxide. In accordance with this, our Janus Pt-MSN nanomotors would be an useful antioxidant therapeutic tool to prevent development and recurrence of tumors and overcome drug resistance. We envision that combining the versatility of stimuli-responsive gatekeepers and catalytic nanomotors can lead to promising advances in nanorobotics and biomedicine in the near future.

## Methods

### Chemicals

Dihydrogen hexachloroplatinate (H_2_PtCl_6_), polyvinylpyrrolidone (PVP), ascorbic acid, 3-mercaptopropionic acid, tetraethyl orthosilicate (TEOS), (3-mercaptopropyl)-trimethoxysilane, *n-*cetyltrimethylammonium bromide (CTABr), paraffin wax, tris(2,2′- bipyridyl)dichlororuthenium(II) hexahydrate (Ru(bpy)_3_Cl_2_), 2,2′-dipyridyl disulfide, *O*-(2-mercaptoethyl)-*O*′-methylhexa(ethylene glycol) (PEG-SH), doxorubicin (C_27_H_29_NO_11_), 2,2′-azinobis(3-ethylbenzothiazoline)-6-sulfonic acid (ABTS), fetal bovine serum (FBS), RPMI-1640 medium, l-glutathione reduced (GSH), Hoechst 33342, and WGA were purchased by Sigma-Aldrich. Sodium hydroxide (NaOH), hydrogen peroxide (30%), sodium hydrogen phosphate monohydrate, disodium hydrogen phosphate heptahydrate, absolute ethanol, toluene, chloroform, and acetonitrile were provided by Scharlau.

### General Methods

TEM images were achieved using a JEOL TEM-2100F electron microscope, and TEM images of cells were acquired using a FEI Tecnai Spirit G2 microscope. STEM-EDX was performed using a JEM 2100F instrument. PXRD measurements were carried out using a Seifert 3000TT diffractometer using Cu Kα radiation. DLS experiments were performed using a ZetaSizer Nano ZS (Malvern). N_2_ adsorption–desorption isotherms were recorded using a Micromeritics TriStar II Plus automated analyzer. Thermal analysis was performed with a TA Instruments SDTQ600 apparatus (USA). Nanoparticle tracking experiments were carried out using a Nanosight NS300 (Malvern). UV–visible measurements were recorded with a JASCO V-650 spectrophotometer. Confocal microscopy imaging was recorded with a Leica TCS SP8 AOBS inverted laser scanning confocal microscope and an XC450 microfluidic device from XonaChip.

### Synthesis of Mesoporous Silica Nanoparticles

To synthesize mesoporous silica nanoparticles, CTAB (1.00 g, 2.74 mmol) was first dissolved in deionized water (480 mL), and the solution was magnetically stirred. Then, to increase the pH, NaOH (3.5 mL, 2 M) was added, followed by an adjustment in the temperature to 80 °C. TEOS, used as inorganic silica precursor, was added dropwise to the surfactant solution. The mixture was stirred for 2 h at 80 °C, giving a white precipitate. Finally, the solid was isolated by centrifugation, washed with deionized water until reaching neutral pH, and dried overnight at 70 °C. The nanomaterial, named “as made”, was calcined at 550 °C using an oxidant atmosphere for 5 h in order to remove the template phase, obtaining the final mesoporous nanomaterial (MSNs).

### Synthesis of Platinum Nanodendrites.^66,67^

For the preparation of platinum nanodendrites, H_2_PtCl_6_ (164 mg) and PVP (20 mg) were dissolved in deionized water (20 mL). Then, a solution of ascorbic acid in water (350 mg in 10 mL) was added drop by drop to the H_2_PtCl_4_ solution. The reaction was heated at 45 °C for 1 h under magnetic stirring. The initially pale yellow color changed to black when the platinum nanoparticles were synthesized.

### Synthesis of Janus Pt-MSN Nanoparticles (**S**_**0**_)

Janus Pt-MSNs were synthesized following an adapted method previously reported by Villalonga *etal*.^45^ Mesoporous silica nanoparticles (180 mg) were dispersed in an aqueous solution (10 mL, 6.7% ethanol), and CTAB was added (208 μL, 1 μM). The temperature of the mixture was increased at 75 °C, and 1 g of paraffin wax was added. When the paraffin was melted, the mixture was homogenized using an Ultra-Turrax T-8 (IKA, Germany) for 10 min. Then, the reaction was further magnetically stirred at 75 °C for 1 h, to form a Pickering emulsion. The result was cooled to room temperature, diluted with methanol (10 mL), and treated with (3-mercaptopropyl)trimethoxysilane (200 μL). The reaction was magnetically stirred for 3 h, and the resultant solid was isolated by centrifugation and washed with methanol two times. Afterward, the partially mercapto-functionalized mesoporous silica nanoparticles were dispersed into the platinum nanoparticles (30 mL). The reaction was stirred overnight at room temperature. Lastly, the solid was filtered, washed with chloroform, and dried. This process yielded the Janus Pt-MSN nanoparticles (**S**_**0**_).

### Synthesis of Janus Pt-MSN (Ru)-PEG (**S**_**1**_)

The platinum face was functionalized with 3-mercaptopropionic acid in order to protect it; 50 mg of **S**_**0**_ was reacted with 70 μL of this molecule for 1 h, using ethanol as solvent. After 1 h, the solid was washed with ethanol three times. The next step was loading the pores with the dye, and the nanoparticles were resuspended in 3 mL of a concentrated solution of [Ru(bpy)_3_]Cl_2_·6H_2_O (30 mg) in anhydrous acetonitrile and stirred for 24 h. Next, we proceeded to functionalize the mesoporous face. First, the solid reacted with 93 μL of (3-mercaptopropyl)trimethoxysilane for 5.5 h. Second, 110 mg of 4,4′-dipyridyl disulfide was added to the mixture, and the reaction was magnetically stirred for 24 h. Third, the thiolated solid was reacted overnight with 50 μL of PEG-SH after being washed with acetonitrile and resuspended in 3 mL of acetonitrile. Finally, the suspension was centrifugated, washed with acetonitrile, and dried under vacuum, yielding the final solid **S**_**1**_.

### Synthesis of Janus Pt-MSN (Doxorubicin)-PEG (**S**_**2**_)

To synthesize this solid, the same procedure used for the preparation of **S**_**1**_ was followed. However, in this case the pores were loaded with the anticancer drug doxorubicin. For 50 mg of **S**_**0**_ we added 24 mg (0.044 mmol) of doxorubicin diluted in 50 mM sodium phosphate buffer pH 7.5. The filling process was carried out in the dark, under magnetic stirring for 24 h. Lastly, once the “molecular gate” was formed, the solid was further washed with 50 mM sodium phosphate buffer pH 7.5. The resulting nanoparticles (**S**_**2**_) were stored in buffer solution at 4 °C.

### Peroxidase-like Activity Assay

To confirm the catalytic activity of our nanomotors, peroxidase activity assays were performed.^68^ The method employed is based on the platinum capability to oxidize ABTS in the presence of H_2_O_2_, mimicking the peroxidase catalytic action. The result is a blue-green product (ABTS*) that can be detected by UV–visible spectrophotometry (λ_abs_ = 405 nm). This reaction is resumed in the next equation:
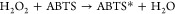


For this assay, dissolutions of ABTS (9 mM) and H_2_O_2_ (from 0 to 30 mM) were prepared in 100 mM PBS pH 5. Afterward, 2.9 mL of ABTS and 0.1 mL of H_2_O_2_ were mixed in a cuvette at 25 °C, and 0.05 mL of **S**_**1**_ (0.5 mg mL^–1^) was added to them. Finally, the variation in the absorbance was recorded for 3 min.

### Motion Analysis by MSD Calculation

To evaluate the nanomotors’ movement, we analyzed the trajectories of single nanoparticles by NTA using a Nanosight NS300 device This technique is based on the tracking of the light scattered by the nanoparticles when a laser beam falls upon them. The device is formed by an sCMOS camera coupled to an optical microscope and a single mode laser diode with 55 mW blue light illumination.

The samples were diluted in sodium phosphate buffer at a concentration of 0.002 mg mL^–1^, ultrasonicated, and introduced into the Nanosight chamber at 25 °C using a 1 mL syringe. Nanomotor motion was registered in five videos of 30 s with a speed of 30 frames s^–1^. The *x*–*y* coordinates of 20 nanoparticles throughout that time (selected size between 100 and 200 nm to avoid aggregates) were extracted by the NTA 3.0 software. That allows the estimation of the MSD for each nanoparticle, obtained applying eq 1 (assuming the motion to be two-dimensional).^16^
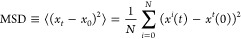
1(*N*: number of averaged nanoparticles; *x*^*i*^ and *x*^*t*^ vector positions of particles at different times).

For the nanoparticles with Brownian motion, the rotational diffusion coefficient was obtained from the plot of MSD *vs* Δ*t*, through the Stokes–Einstein equation (MSD = 4*D*_r_Δ*t*), whereas for the nanoparticles that follow a directional motion, the parabolic component of the Stokes–Einstein equation (MSD = 4*D*_t_Δ*t* + (*v*Δ*t*)^2^) is applied to obtain the velocity and translational diffusion coefficient.

The time interval used in this study was set below the rotational diffusion time (τ_r_) where propulsion movement dominates, theoretically calculated using the following equation:
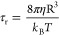
2where η is the viscosity (10^–3^ Pa s), *k*_B_ is the Boltzmann constant, *T* is the temperature (25 °C), and *R* is the radius of the particle (145 nm, assuming a spherical morphology). Under these conditions a value of 0.8 s was obtained for τ_r_.

To calculate the propulsion force (*F*_Prop_) or the drag force (*F*_drag_) applied on activated nanomotors, we employed the drag law of Stokes in fluids, assuming the nanomotor morphology as spherical.
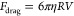
3where η is the viscosity (10^–3^ Pa) and *R* is the radius of the particle (145 nm). Calculated *F*_drag_ were 5.6, 8.5, 16, and 53 fN for 0.02%, 0.7%, 0.2%, and 0.35% of fuel concentration, respectively.

### Control Release Studies

Release of Ru(bpy)_3_Cl_2_ from **S**_**1**_ was carried out under static and stirring conditions, to study the effect of the nanomotor self-propulsion on the delivery of the cargo. In a typical stirring experiment, four different suspensions of 0.5 mg of **S**_**1**_ in 1 mL of 50 mM PBS (pH 7.5) were prepared. Solutions were placed in a shaker (Thermo-shaker HC24N Grant Instruments PCMT) for 30 min at 14 000 rpm. After this period, H_2_O_2_, GSH, or both were added at final concentrations of 1% and 10 mM, respectively (registered as zero release time). At scheduled times (2, 5, 10, 15, 20, and 30 min), 200 μL aliquots were taken and centrifugated (3 min, 125 000 rpm) to isolate the Ru(bpy)_3_Cl_2_ released in the supernatant from the nanoparticles. Then, fluorescence emission of the dye was monitored at 595 nm with a JASCO spectrofluorometer FP-8300. The results of three independent experiments are shown in Figure SI-7A. In the static experiment, the four samples were prepared as previously described (0.5 mg mL^–1^ in 50 mM PBS, pH 7.5) but remained without external stirring. After 30 min, the inputs (H_2_O_2_, GSH, or both, at final concentrations of 0.1% and 10 mM, respectively) were carefully added through the wall of the tubes containing the samples. Afterward, 200 μL aliquots were taken at different times (2, 5, 10, 15, 20, and 30 min) and centrifuged (3 min, 125 000 rpm) to sediment the nanodevices. Fluorescence of Ru(bpy)_3_Cl_2_ delivered to the media was measured at 595 nm (λ_exc_ = 453 nm), Figure 4.A. The doxorubicin release from the **S**_**2**_ assay was further performed in static conditions, following the same procedure as for **S**_**1**_. In this case, the doxorubicin fluorescence emission was monitored at 555 nm (λ_exc_ = 470 nm). The kinetic curves of four independent experiments are shown in Figure SI-7B.

### Cell Culture Conditions

THP-1 cells, a human monocytic cell line derived from an acute monocytic leukemia patient, were purchased from ATCC and were grown in RPMI-1640 supplemented with 10% FBS. Cells were incubated at 37 °C in an atmosphere of 5% carbon dioxide.

### Viability Assay

THP-1 cells were seeded in 96-well plates at 800 000 cells/well and incubated at 37 °C for 24 h. Then, cells were treated with different reagent concentrations to evaluate their toxicity to cells. The reagents analyzed were H_2_O_2_, **S**_**2**_ nanomotors, and free doxorubicin. Finally, cell viability was evaluated by incubation with the cell proliferation WST-1 reagent for 1 h and measuring the absorbance at 595 nm.

### Cell Uptake of Nanomotors by Confocal Microscopy

THP-1 cells were seeded at 10^6^ cells mL^–1^ in a six-well plate and incubated with **S**_**2**_ nanomotors (50 μg mL^–1^) in RPMI-1640 buffer supplemented with 10% FBS at 37 °C for 30 min in the presence and absence of 0.02% (8.5 mM) H_2_O_2_. Afterward, uninternalized nanoparticles were removed from cells by a washing step and further incubated for 1, 4, and 6 h in a fresh media. Next, cells were seeded on glass coverslips, and DNA and membrane markers, Hoechst 33342 and WGA (wheat germ agglutinin), respectively, were added. Finally, slides were visualized under a Leica TCS SP2 AOBS inverted laser scanning confocal microscope (Leica Microsystems Heidelberg GmbH, Mannheim, Germany). The images were acquired with an excitation wavelength of 405 nm for Hoescht, 480 nm for doxorubicin, and 650 nm for WGA. The distribution of fluorescence inside the cells was analyzed using ImageJ software (Figure 6A).

### Cell Uptake of Nanomotors by TEM

THP-1 cells were seeded in a six-well plate at 800 000 cells/well and incubated at 37 °C for 24 h. Then, cells were treated with Janus nanomotors (100 μg mL^–1^) at 37 °C for 6 h. After this period, cells were washed with 0.1 M phosphate buffer (pH 7.4) and fixed in a 2.5% glutaraldehyde + 2% paraformaldehyde solution for 1 h at room temperature and 72 h more at 4 °C. Fixed cells were washed with phosphate buffer, dehydrated in ethanol, and stained with uranyl acetate (1%) and osmium tetroxide (1%). Finally, samples were included in epoxy resin (Araldite) and sectioned for TEM analysis. TEM images were acquired using a FEI Tecnai Spirit G2 microscope operating at 80 kV with a digital camera (Soft Image System, Morada), Figure 6C.

### Self-Propulsion and Drug Release Capabilities of **S**_**2**_ in Microchip Device

Movement and delivery abilities of nanomotors were performed by recording confocal microscopy videos using a four-compartment microchip device (acquired from XonaChips) with a 150 μm microgroove barrier and a 75 × 25 mm size. This device has four wells connected two by two (1–2; 3–4), and these were connected to each other by micrometric channels (Figure 6). The experiment was carried out by depositing 106 THP-1 cells/mL in wells 1 and 2 dyed with WGA and 100 μg mL^–1^ of **S**_**2**_ nanomotors in position 4 and completing the volume with RPMI-1640 buffer in well 3 (final volume 150 μL per well). At this point, the video was started after a few minutes, in order to eliminate the diffusive movement of the nanomotors after their addition to the well. Brownian motion of nanodevices was then recorded using a Leica TCS SP2 AOBS confocal microscope during 5 min. Then, the same methodology was repeated adding the fuel (H_2_O_2_) in position 3 to a total concentration of 0.02% in the cuvette (fuel volume added was previously removed from well 3 to maintain a constant total volume). Furthermore, to study the controlled doxorubicin release from **S**_**2**_ in the cells, a previous experimental procedure was also followed but for a prolonged time of 30 min. In the recorded videos, assembled nanomotors (up to 350 nm) can be observed through the excitation of the doxorubicin loaded in the silica nanopores because of the confocal microscopy resolution.^69^ Finally, nanomotor trajectories were drawn with the plugin Manual Tracking from ImageJ software,^70^ and the intensity of cellular fluorescence was also analyzed using LAS X software and the “Line Profile” tool. This tool measures the fluorescence intensity along the linear regions of interest (ROI) defined by us and are shown graphically as a curve. Five regions of interest were defined (ROI 01–05), and the fluorescence intensity associated with doxorubicin was analyzed in each condition. Line profiles were placed through the cells, and the peak currents were evaluated simultaneously at time 0 and 30 min (Figure 7).

## Supporting Information Available

H_2_O_2_-fueled motors’ comparison (Tables SI 1, 2), TEM images, hydrodynamic diameter, zeta potential, PXRD patterns, N_2_ adsorption–desorption isotherms, activity assay, images of bubble growth and zoomed trajectories, diffusion coefficients, release capability in stirring conditions, and doxorubicin release kinetics (PDF)
Video SI-1 of nanomotor trajectory tracking (complete assay, 2 min) (AVI)
Video SI-2 of nanomotor trajectory tracking (without fuel, 0–20 s) (AVI)
Video SI-3 of nanomotor trajectory tracking (with 0.02% fuel concentration, 20–40 s) (AVI)

## Supplementary Material

nn0c08404_si_001.pdf

nn0c08404_si_002.avi

nn0c08404_si_003.avi

nn0c08404_si_004.avi
