# Supplementary material for: Ultrafast Directional Janus Pt–Mesoporous Silica Nanomotors for Smart Drug Delivery
Source: ACS Nano. 2021 Mar 6;15(3):4467–80. doi: 10.1021/acsnano.0c08404 (PMC8719758; doi:10.1021/acsnano.0c08404)
Supplement: Supplementary file 1 — nn0c08404_si_001.pdf [file nn0c08404_si_001.pdf]

Supporting Information

Ultra-Fast Directional Janus Pt-Mesoporous Silica Nanomotors for Smart Drug Delivery

Paula Díez, [abd] *Elena Lucena-Sánchez,* [abd] *Andrea Escudero,* [abd] *Antoni Llopis-Lorente,* [abd] *Reynaldo Villalonga,* [e] *Ramón Martínez-Máñez** [abcd]

[a] Instituto Interuniversitario de Investigación de Reconocimiento Molecular y Desarrollo Tecnológico (IDM), Universitat Politècnica de València, Universitat de València, Spain, Camino de Vera s/n, 46022 València, Spain.

[b] Unidad Mixta UPV-CIPF de Investigación en Mecanismos de Enfermedades y Nanomedicina, Valencia, Universitat Politècnica de València, Centro de Investigación Príncipe Felipe, 46012 València, Spain.

[c] Unidad Mixta de Investigación en Nanomedicina y Sensores. Universitat Politècnica de València, Instituto de Investigación Sanitaria La Fe, 46026 Valencia, Spain.

[d] CIBER de Bioingeniería, Biomateriales y Nanomedicina (CIBER-BBN), 28029 Madrid, Spain.

[e] Nanosensors & Nanomachines Group, Department of Analytical Chemistry, Faculty of Chemistry, Complutense University of Madrid, 28040, Madrid, Spain.

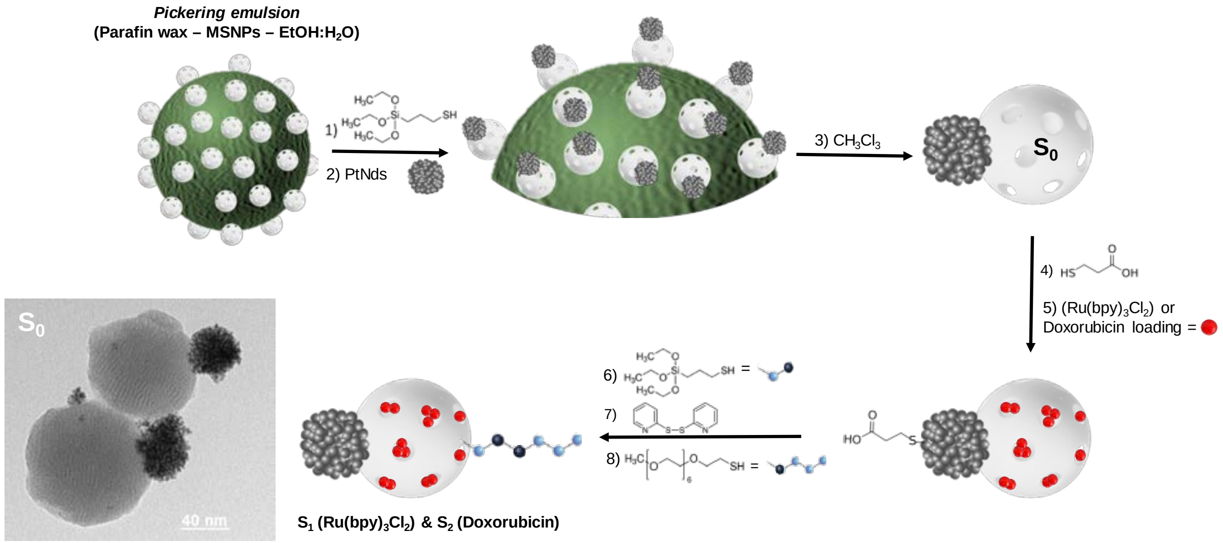

SI-Scheme 1. Schematic Illustration of the steps in the preparation of Janus Pt-MSN nanomotors. TEM image of representative Janus Pt-MSN nanomotors (**S_0**).

Table SI-1. Representative published H_2O_2-fueled nano- and micro-motors. i) Janus Pt-MSN nano and micromotors. ii) nanomotors with other designs. Most of the described motors need high amounts of fuel and does not achieve directional movement.

Motor type	Size	$[H_2O_2]$ (%)	Velocity	Reference
**i) Janus Pt-MSN**				
**NANOMOTORS**				
**Pt-MSN**	**145 nm**	**0.3**	**19.4 µm s^{-1}**<br>**461 body length s^{-1} $\%^{-1}$**	**(Present work)**
Pt/Cr-MSN	75 nm	30	6 mm s^{-1}<br>8 body length s^{-1} $\%^{-1}$	27
Heparin-Pt -HMS	250 nm	15	212 mm s^{-1}<br>56.5 body length s^{-1} $\%^{-1}$	26
Pt-SiO_2	480 nm	15	5.6 mm s^{-1}<br>0.8 body length s^{-1} $\%^{-1}$	11
Pt- HMS	500 nm	1.5	D = 1.49 µm^2 s^{-1}<br>(diffusion coefficient)	16
Pt-MSN	<100 nm	2.5	D = 9.20 µm^2 s^{-1}	28

MICROMOTORS				
Coloid Co/Pt-SiO_2	5 mm	15	9 mm s^{-1}<br>0.9 body length s^{-1} $\%^{-1}$	29
Pt- SiO_2	5 mm	5	2.5º μm^{-1} (angular speed)	30
Pt-SiO_2 beads	3 mm	5	0.41 mm s^{-1}<br>0.027 body length s^{-1} $\%^{-1}$	31
Pt/Au-SiO_2	2 mm	5	D = 0.88 μm^2 s^{-1}	32
Pt-SiO_2	2.5 mm	3	6 mm s^{-1}<br>1.33 body length s^{-1} $\%^{-1}$	33
**ii) Other designs**				
InGaAs/ GaAs/(Cr)Pt nanotubes	280-600 nm	20	110 mm s^{-1}<br>18.3 body length s^{-1} $\%^{-1}$	34
Pt Nanoparticle - Antibody	20 nm	10	1.2 mm s^{-1}<br>5.99 body length s^{-1} $\%^{-1}$	35
Janus Pt-VLP NM viral	25-30 nm	1.5	4.15 mm s^{-1}<br>110.67 body length s^{-1} $\%^{-1}$	36
(PEG-PS) polymersome - based Janus	100-300 nm	1	D = 3.7 μm^2 s^{-1}	37
Polymer stomatocytes - Pt NP	316 nm	0.3	23 mm s^{-1}<br>242.6 body length s^{-1} $\%^{-1}$	38

Table SI-2. Main features and innovation points of the presented nanomotors in comparison to previously reported Janus Pt-MSN nanomotors.

Feature	Previous works	Present work	Provided advantages
**Catalytic component**	Metallic layer	Pt nanodentrites	Higher catalytic surface
**Architecture**	Half-coated Janus	Snowman-like Janus	Minimal reduction of effective silica surface, catalytic component location

**Fabrication method**	Electron beam evaportation/sputtering	Pickering emulsion	Higher yield, no need of expensive equipment
**Controlled release**	No	Yes	Reading information from the environment and controlled cargo release
**Fuel concentration**	1-10%	0.02-0.35%	Lower concentrations
**Biological studies**	None/cell culture	Directional motion through capillary and internalization	Demonstration of advanced functionality

1. Nanomaterials Characterization:

The nanodevices $\mathbf{S_0}$, $\mathbf{S_1}$ and $\mathbf{S_2}$ were characterized using standard procedures.

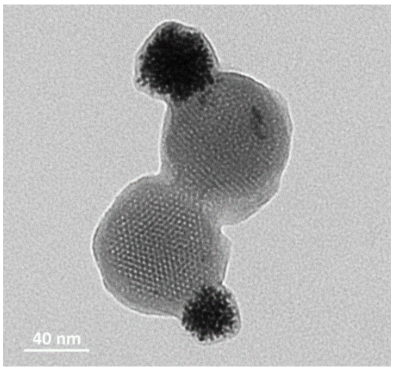

Figure SI-1. TEM image of Janus Pt-MSN nanomotors ($\mathbf{S_0}$) after catalytic reaction in a 30% of H_2O_2 solution for 24 h.

Powder X-ray diffraction analysis

In the **Figure SI-3** are represented the PDRX patterns of the calcined MSNPs $\mathbf{S_0}$ and $\mathbf{S_1}$ at low ($1.5 < 2\theta > 7$) and at high angles ($35 < 2\theta > 80$). $\mathbf{S_0}$ presents a main peak around 2.4°, which is typical of mesoporous materials with MCM-41 structure. This low angle reflection was preserved in $\mathbf{S_1}$, revealing that the processes of cargo loading and chemical functionalization did not transform the mesoporous structure. In addition, at high angles $\mathbf{S_1}$ shows several

diffraction patterns indexed as (111), (200), (220) and (311) Bragg peaks of a Pt cubic structure.

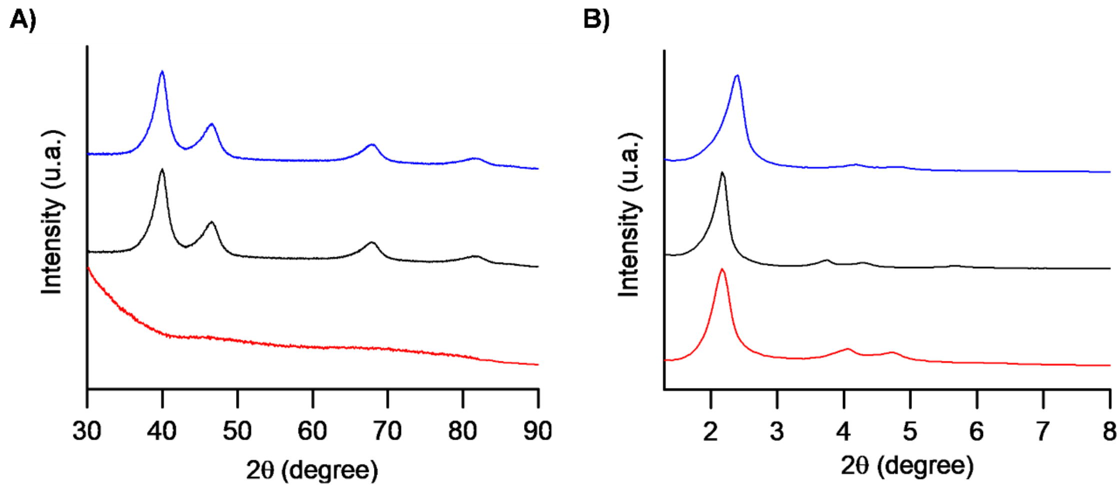

Figure SI-2. PDRX pattern at high-angles (A) and low-angles (B) of calcined MSNs (red curve), **S_0** (black curve) and **S_1** (blue curve).

<u>N_2 adsorption-desorption isotherms</u>

The N_2 adsorption-desorption isotherms of **S_0** and **S_1** are shown in **Figure SI-4**. **S_0** presents two principal adsorption steps. Between 0.2 and 0.4 P/P_0 value shows the first step, which is due to the nitrogen condensation inside the mesopores by capillarity and reveals the emptiness of the pores. Besides, the lack of hysteresis loop suggest that the pores are cylindrical and uniform. The second step at P/P_0 value around 0.9 shows a typical H1 hysteresis loop. It is considered as textural like porosity and correspond to the space among nanoparticles. In contrast, the N_2 adsorption-desorption isotherm of **S_1** lacks the sharp step at low-medium relative pressure, exhibiting an important decrease in the N_2 volume adsorbed, which indicates that the loading and capping processes have been successful.

Total pore volume and pore size were estimated by using the Barret, Joyner an Halenda (BJH) model on the adsorption branch of the isotherm, for $P/P_0 < 0.4$, which is associated to the surfactant generated mesopores. Brunauer, Emmett and Teller (BET) specific values are

calculated from N_2 adsorption-desorption isotherms and show an important reduction in **MSN** **(**1068 $m^2 \cdot g^{-1}$) compared to **S_0** (432 $m^2 \cdot g^{-1}$), which is due to the attachment of non-porous platinum nanoparticles. Moreover, the surface reduction is even superior in **S_1** upon cargo loading and functionalization with the gating system (25 $m^2 \cdot g^{-1}$) Both, BJH and BET values are shown in Table SI-1.

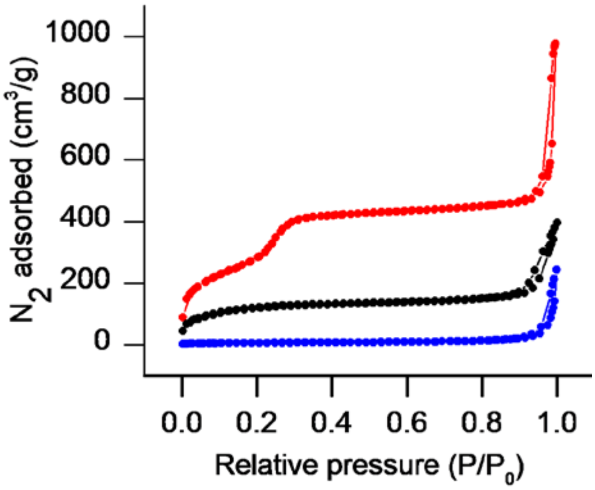

Figure SI-3. N_2 adsorption-desorption isotherms for calcined MSNPs (red curve), starting Janus Pt-MSNPs **S_0** (black curve) and final Janus Pt-MSNPs **S_1** (blue curve) (A), and corresponding pore diameter distribution (BJH model) (B).

Table SI-3. Pore diameters, pore volumes and BET specific surface values calculated from the N_2 adsorption-desorption isotherms for prepared nanomaterials.

	BJH pore (P/P_0 < 0.4) (nm)	Total pore volume ($cm^3 \cdot g^{-1}$)	S_{BET} ($m^2 \cdot g^{-1}$)
MSNs	1.96	0.96	1068
S_0	1.97	0.38	432
S_1	-	-	25

Thermogravimetric assay

Thermogravimetric studies of **S_0** and **S_1** are shown in **Figure SI-4** reveal the organic content corresponding to the capping system (S-S-PEG) and the organic content of the loaded $Ru(bpy)_3Cl_2$. The first thermal decomposition above ~245 ºC corresponds to the gatekeeper, while the second one at ~ 320 ºC is attributed to the loaded dye.

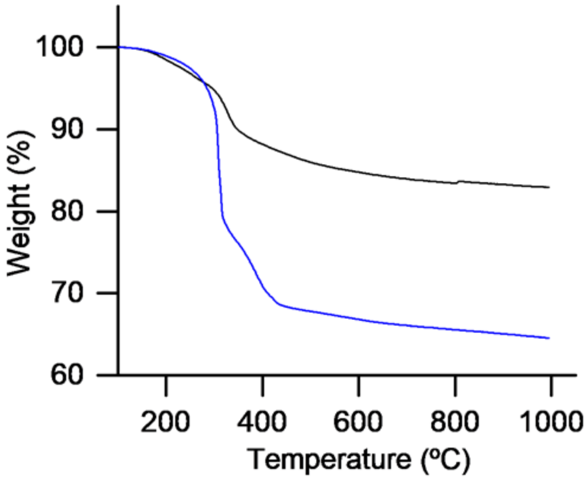

Figure SI-4. Thermogravimetric analysis of **S_0** (black curve) and **S_1** (blue curve).

DLS analysis

The hydrodynamic size and zeta potential of **S_0** and **S_1** were determined by dynamic light scattering studies. To that purpose, the solids were suspended in distilled water at a concentration of 0.01 mg mL^{-1}. The results, shown in **Figure SI-5**, reveal a decrease in negative surface charge as well as an increment in the hydrodynamic diameter, both as a consequence of molecular gate (S-S-PEG) attachment and cargo loading in the mesoporous face of the nanomotor.

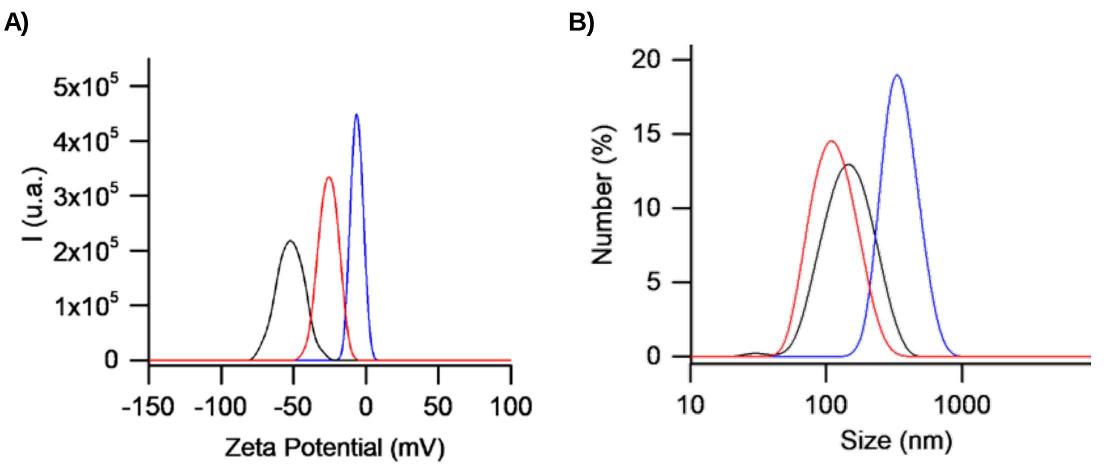

Figure SI-5. DLS analysis showing the zeta potential (A) and hydrodynamic diameter (B) of calcinated MCM-41 (red curve), $\mathbf{S_0}$ (black curve) and $\mathbf{S_1}$ (blue curve) nanomaterials.

2. Motion analysis

Peroxidase-like activity assay

The nanomotors present a typical Michaelis-Menten kinetic. The values of the Michaelis constant (K_M) and the maximum velocity (V_{max}), defined as the maximum rate at which an enzyme can catalyze a reaction, were estimated from the Lineweaver-Burk graphs. Data show that the nanomotor owns an intrinsic peroxidase activity. For a variable concentration of H_2O_2, K_M was 2.3 mM and V_{max} was 0.32 μM min^{-1}.

Equation SI-1:

$$\frac{1}{v} = \frac{K_M}{v_{max}} \cdot \frac{1}{[S]} + \frac{1}{v_{max}}$$

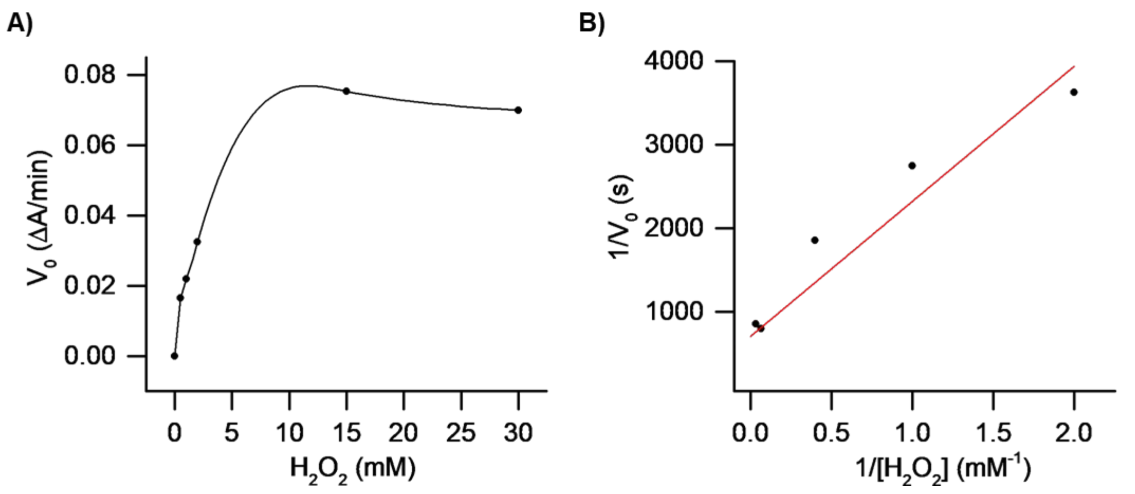

Figure SI-6. Peroxidase-like activity assay: effect of H_2O_2 concentration on the nanomotor catalyzed reaction rate at fixed ABTS dye concentration in 9 mM and temperature at 25°C (A). Lineweaver-Burk plot of H_2O_2-catalysed decomposition used to determinate K_M applying the **equation SI-1** (B).

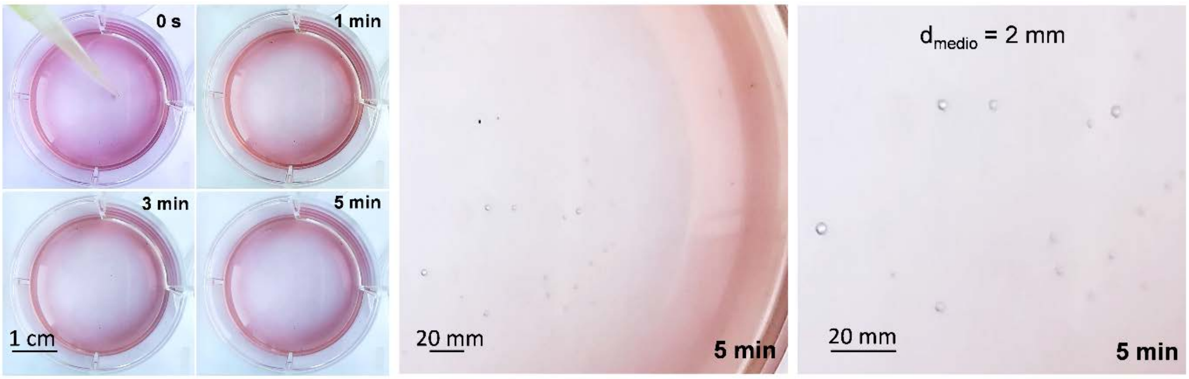

Figure SI-7. Time-lapse images of bubble growth by H_2O_2-propelled **S_1** nanomotors solutions. Bubble generation (average diameter: 2 mm) by the catalytic decomposition of H_2O_2 can be observed after 5 min of fuel addition.

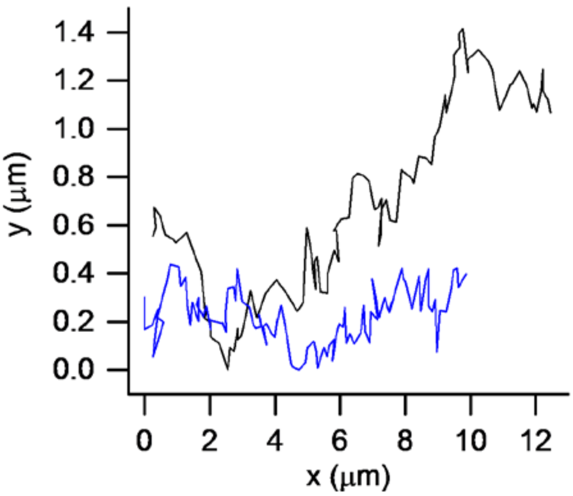

Figure SI-8. Zoomed trajectories (on y-positions) for two Janus Pt-MSN **S1** nanomotors at maximum fuel concentration tested (0.35%), where the random reorientations suffered by nanomotors are observed.

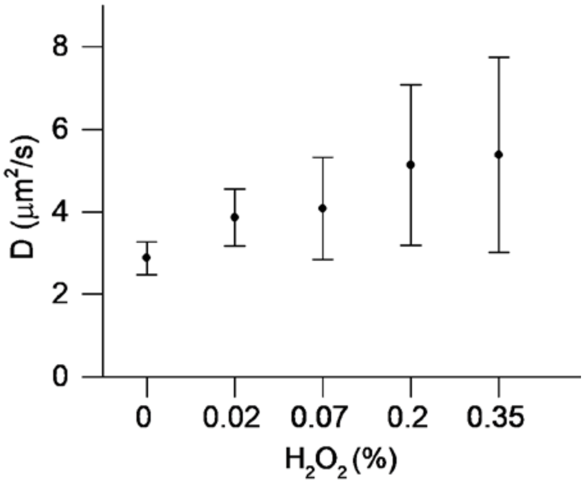

Figure SI-9. Diffusion coefficients determined from the MSD *vs* Δt equations (lineal and parabolic form) for the nanomotors at different fuel concentrations (n= 6).

3. Control release studies

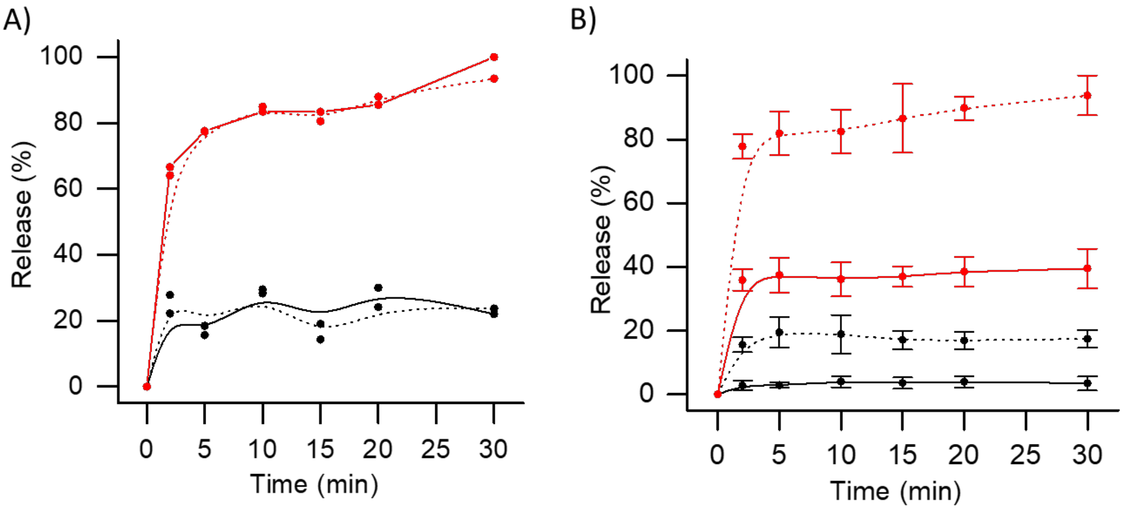

Figure SI-10. Normalized cargo release from Janus Pt-MSN nanomotors **S_1** under stirring conditions determined by measuring $Ru(bpy)_3Cl_2$ fluorescence (at 595 nm) **(A)** and normalized cargo release from nanomotors **S_2** in static conditions determined by measuring Doxorubicin fluorescence (at 555 nm) **(B)** *vs* time in aqueous solution (50 mM PBS pH 7.5), using: (a) nanomotors without GSH and without H_2O_2 addition, (b) 0.1% H_2O_2-propelled nanomotors without GSH addition, (c) nanomotors with 10 mM GSH addition and without H_2O_2, and (d) 0.1% H_2O_2-propelled nanomotors with 10 mM GSH addition.

4. Cells assays

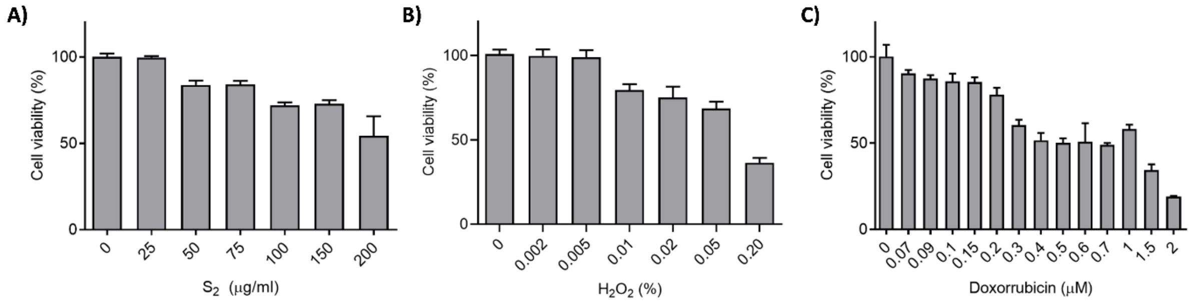

Figure SI-11. Viability of THP-1 cells after 24 h of incubation with **S_2** nanomotors (A), different concentrations of H_2O_2 (from 0 to 0.2%) (B), free Doxorubicin (C).
